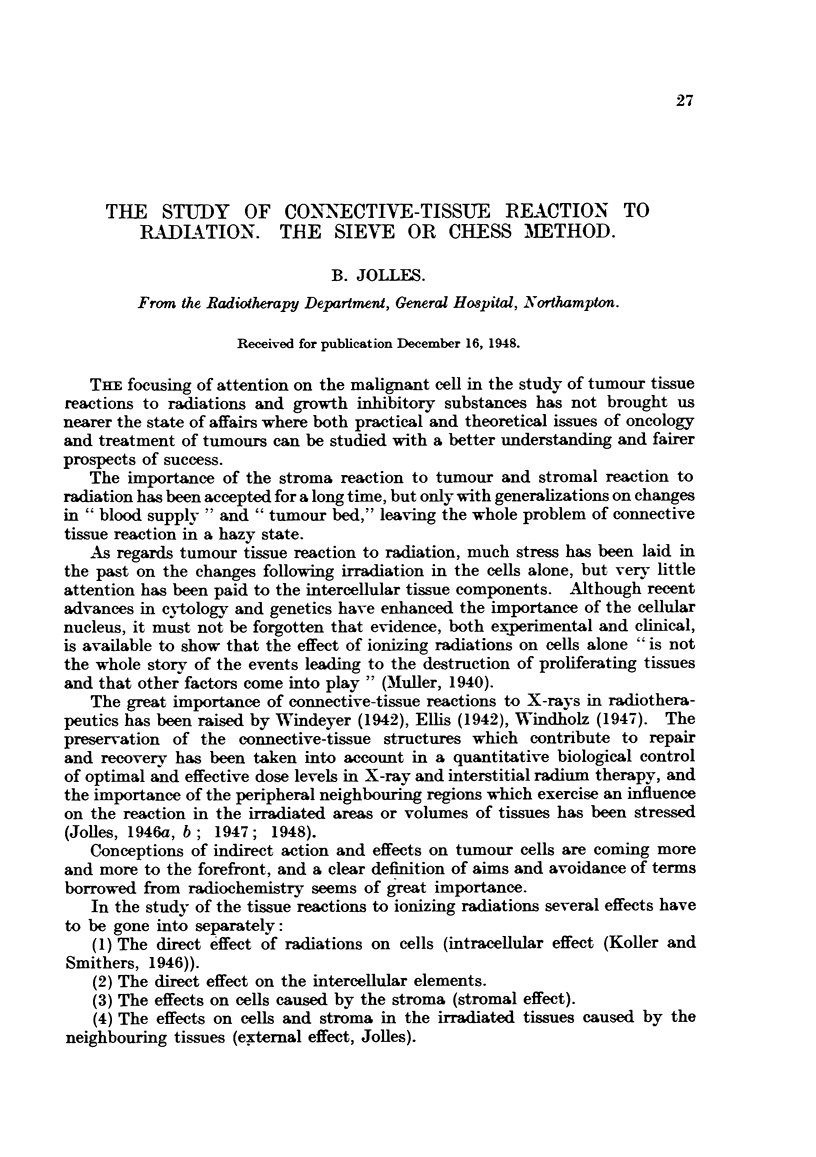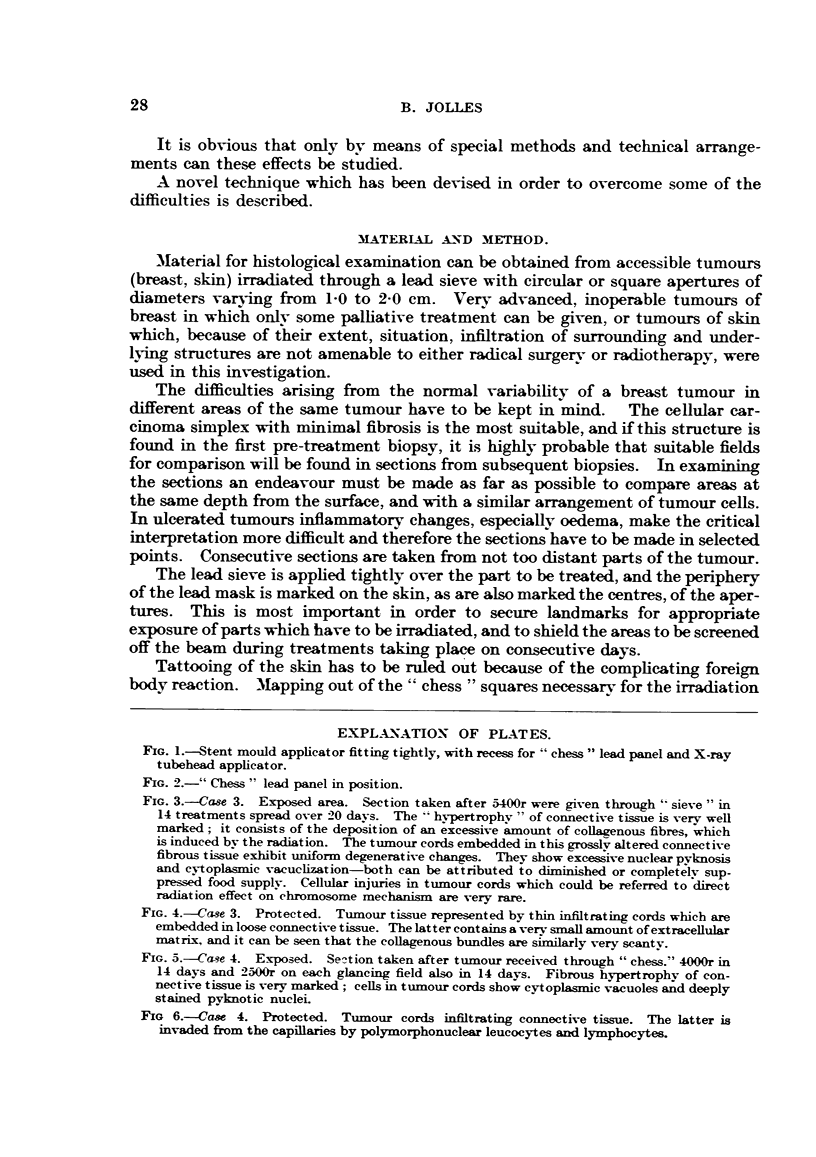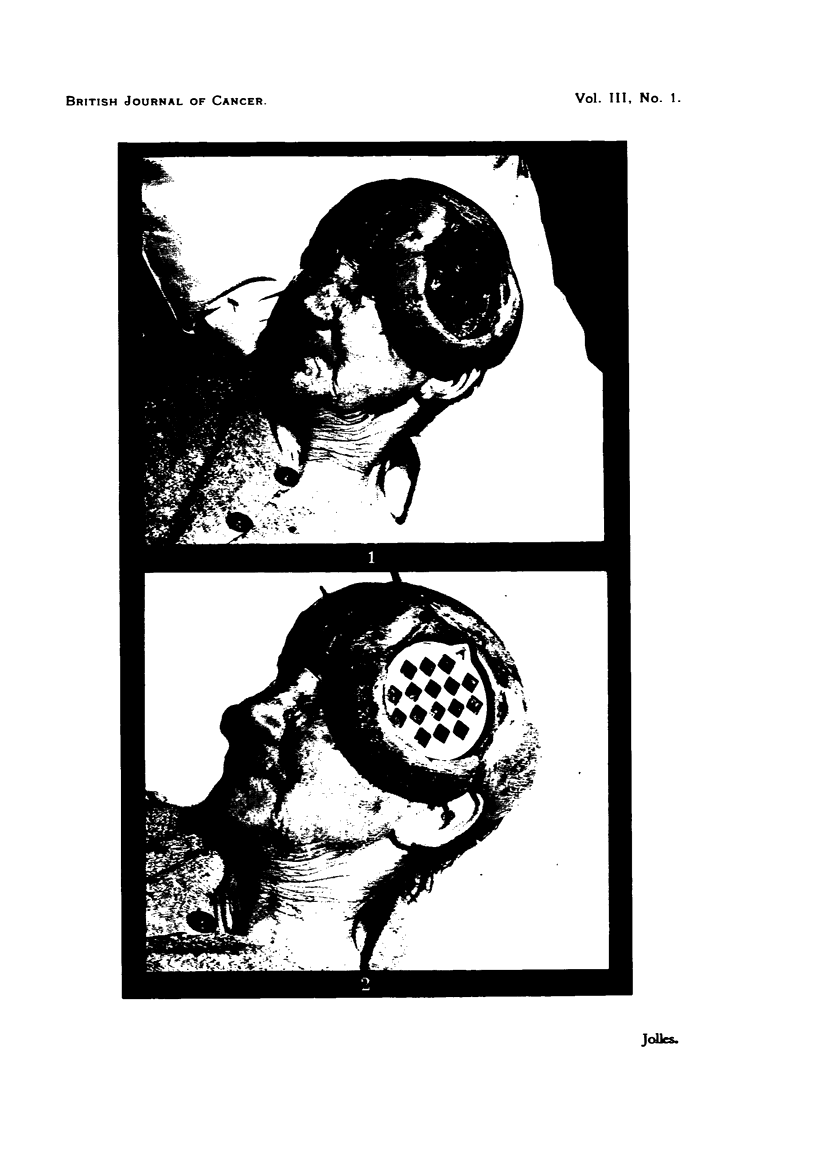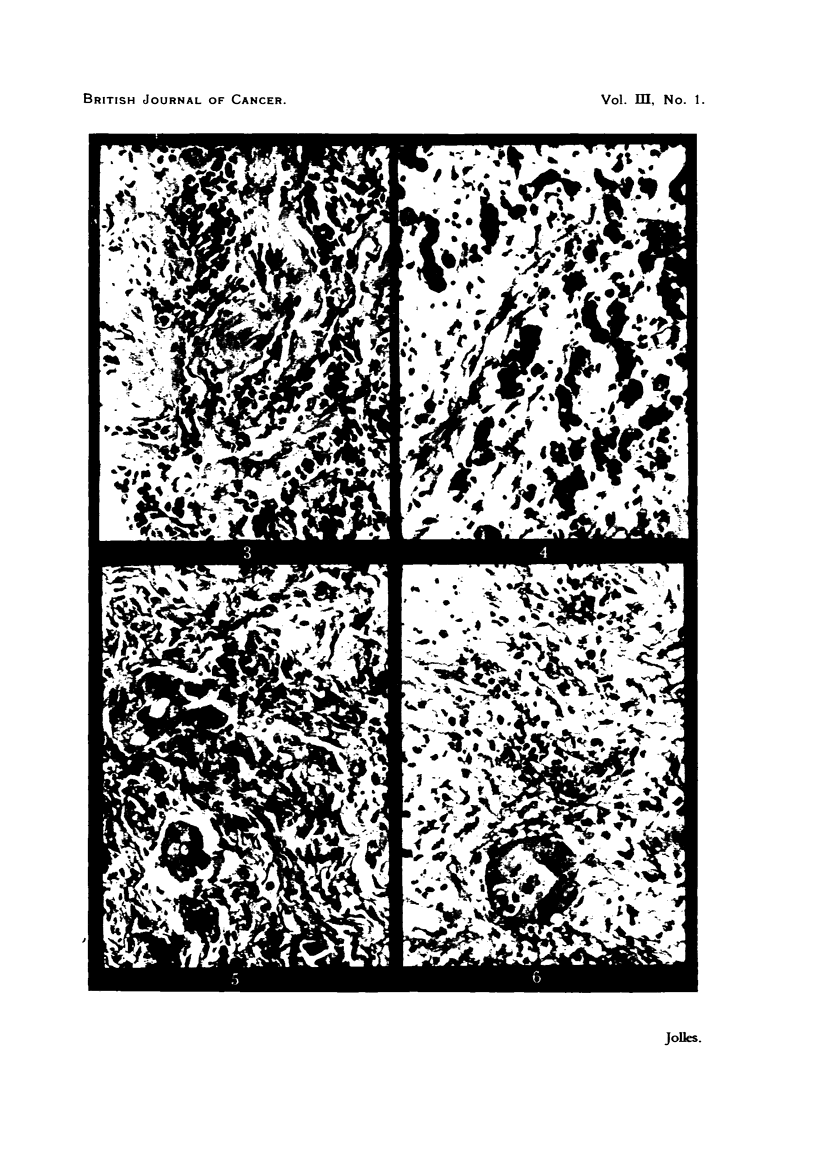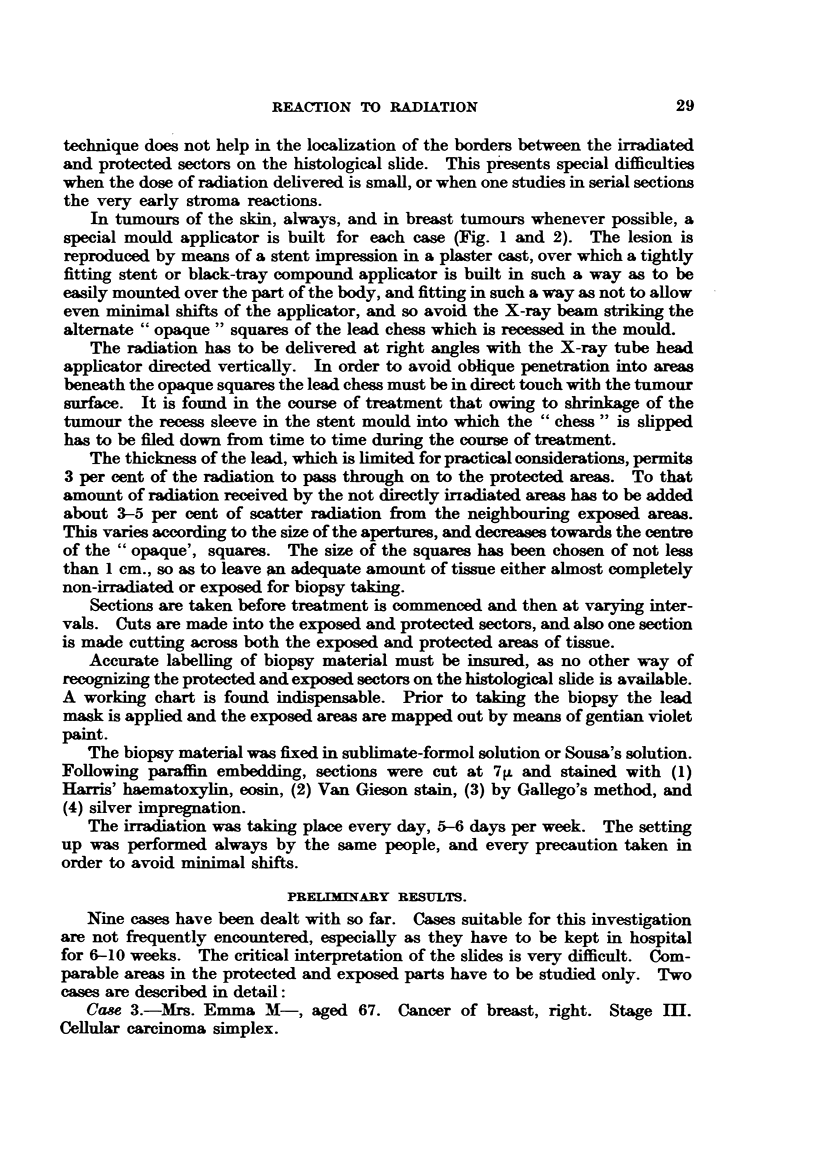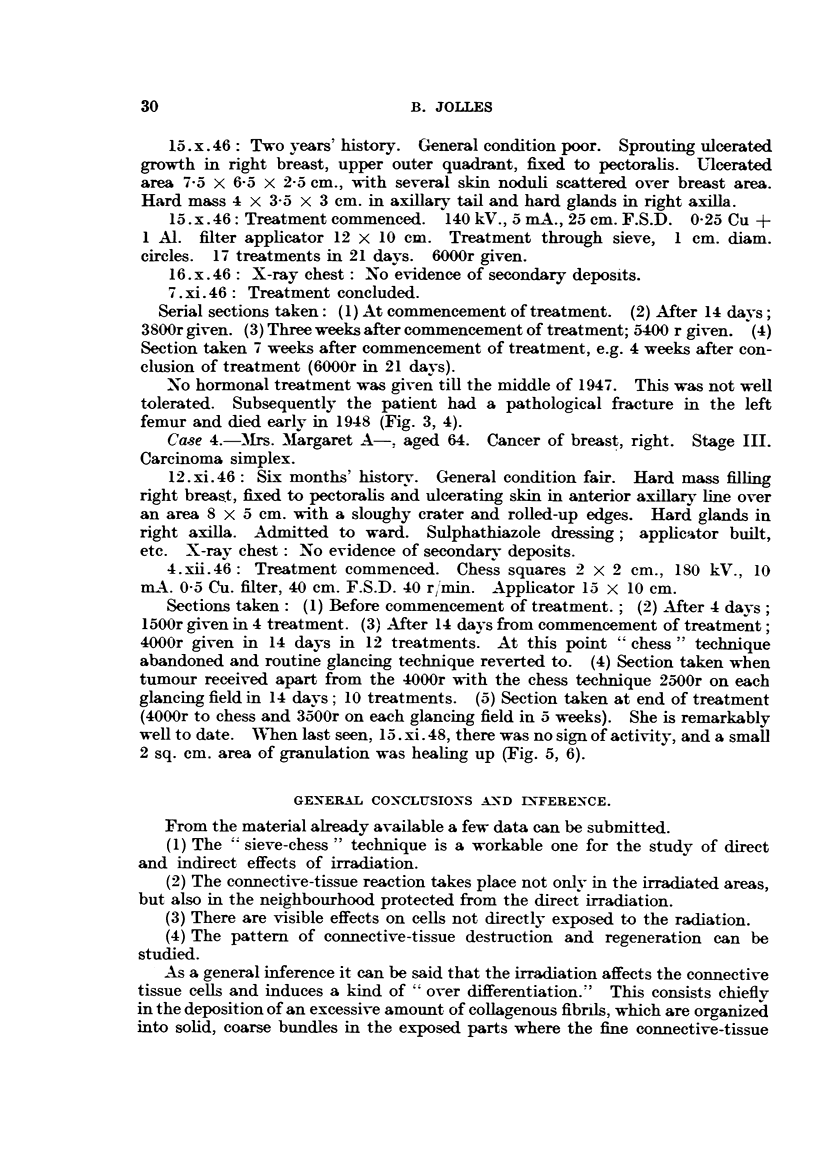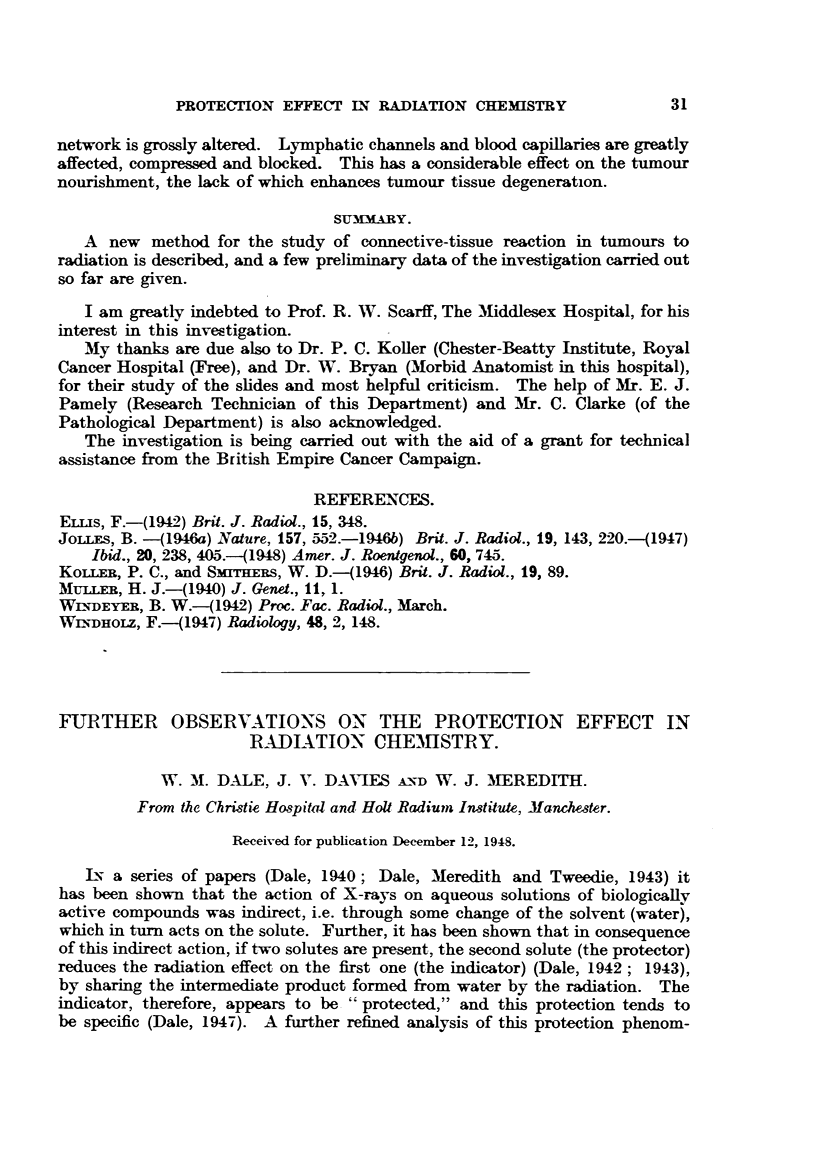# The Study of Connective-Tissue Reaction to Radiation. The Sieve or Chess Method

**DOI:** 10.1038/bjc.1949.3

**Published:** 1949-03

**Authors:** B. Jolles

## Abstract

**Images:**


### THE STUDY OF CONNECTIVE-TISSUE REACTION TO RADIATION. THE SIEVE OR CHESS METHOD.

B. JOLLES.

*From the Radiotherapy Department, General Hospital, Northampton.*

Received for publication December 16, 1948.

THE focusing of attention on the malignant cell in the study of tumour tissue reactions to radiations and growth inhibitory substances has not brought us nearer the state of affairs where both practical and theoretical issues of oncology and treatment of tumours can be studied with a better understanding and fairer prospects of success.

The importance of the stroma reaction to tumour and stromal reaction to radiation has been accepted for a long time, but only with generalizations on changes in " blood supply " and " tumour bed," leaving the whole problem of connective tissue reaction in a hazy state.

As regards tumour tissue reaction to radiation, much stress has been laid in the past on the changes following irradiation in the cells alone, but very little attention has been paid to the intercellular tissue components. Although recent advances in cytology and genetics have enhanced the importance of the cellular nucleus, it must not be forgotten that evidence, both experimental and clinical, is available to show that the effect of ionizing radiations on cells alone " is not the whole story of the events leading to the destruction of proliferating tissues and that other factors come into play " (Muller, 1940).

The great importance of connective-tissue reactions to X-rays in radiotherapeutics has been raised by Windeyer (1942), Ellis (1942), Windholz (1947). The preservation of the connective-tissue structures which contribute to repair and recovery has been taken into account in a quantitative biological control of optimal and effective dose levels in X-ray and interstitial radium therapy, and the importance of the peripheral neighbouring regions which exercise an influence on the reaction in the irradiated areas or volumes of tissues has been stressed (Jolles, 1946*a*, *b* ; 1947 ; 1948).

Conceptions of indirect action and effects on tumour cells are coming more and more to the forefront, and a clear definition of aims and avoidance of terms borrowed from radiochemistry seems of great importance.

In the study of the tissue reactions to ionizing radiations several effects have to be gone into separately:

(1) The direct effect of radiations on cells (intracellular effect (Koller and Smithers, 1946)).

(2) The direct effect on the intercellular elements.

(3) The effects on cells caused by the stroma (stromal effect).

(4) The effects on cells and stroma in the irradiated tissues caused by the neighbouring tissues (external effect, Jolles).

It is obvious that only by means of special methods and technical arrangements can these effects be studied.

A novel technique which has been devised in order to overcome some of the difficulties is described.

### MATERIAL AND METHOD.

Material for histological examination can be obtained from accessible tumours (breast, skin) irradiated through a lead sieve with circular or square apertures of diameters varying from 1·0 to 2·0 cm. Very advanced, inoperable tumours of breast in which only some palliative treatment can be given, or tumours of skin which, because of their extent, situation, infiltration of surrounding and underlying structures are not amenable to either radical surgery or radiotherapy, were used in this investigation.

The difficulties arising from the normal variability of a breast tumour in different areas of the same tumour have to be kept in mind. The cellular carcinoma simplex with minimal fibrosis is the most suitable, and if this structure is found in the first pre-treatment biopsy, it is highly probable that suitable fields for comparison will be found in sections from subsequent biopsies. In examining the sections an endeavour must be made as far as possible to compare areas at the same depth from the surface, and with a similar arrangement of tumour cells. In ulcerated tumours inflammatory changes, especially oedema, make the critical interpretation more difficult and therefore the sections have to be made in selected points. Consecutive sections are taken from not too distant parts of the tumour.

The lead sieve is applied tightly over the part to be treated, and the periphery of the lead mask is marked on the skin, as are also marked the centres, of the apertures. This is most important in order to secure landmarks for appropriate exposure of parts which have to be irradiated, and to shield the areas to be screened off the beam during treatments taking place on consecutive days.

Tattooing of the skin has to be ruled out because of the complicating foreign body reaction. Mapping out of the " chess " squares necessary for the irradiation

---

### EXPLANATION OF PLATES.

FIG. 1.—Stent mould applicator fitting tightly, with recess for " chess " lead panel and X-ray tubehead applicator.

FIG. 2.—" Chess " lead panel in position.

FIG. 3.—*Case* 3. Exposed area. Section taken after 5400r were given through " sieve " in 14 treatments spread over 20 days. The " hypertrophy " of connective tissue is very well marked; it consists of the deposition of an excessive amount of collagenous fibres, which is induced by the radiation. The tumour cords embedded in this grossly altered connective fibrous tissue exhibit uniform degenerative changes. They show excessive nuclear pyknosis and cytoplasmic vacuclization—both can be attributed to diminished or completely suppressed food supply. Cellular injuries in tumour cords which could be referred to direct radiation effect on chromosome mechanism are very rare.

FIG. 4.—*Case* 3. Protected. Tumour tissue represented by thin infiltrating cords which are embedded in loose connective tissue. The latter contains a very small amount of extracellular matrix, and it can be seen that the collagenous bundles are similarly very scanty.

FIG. 5.—*Case* 4. Exposed. Section taken after tumour received through " chess." 4000r in 14 days and 2500r on each glancing field also in 14 days. Fibrous hypertrophy of connective tissue is very marked; cells in tumour cords show cytoplasmic vacuoles and deeply stained pyknotic nuclei.

FIG 6.—*Case* 4. Protected. Tumour cords infiltrating connective tissue. The latter is invaded from the capillaries by polymorphonuclear leucocytes and lymphocytes.

1

2

Jolles.

Jolles.

technique does not help in the localization of the borders between the irradiated and protected sectors on the histological slide. This presents special difficulties when the dose of radiation delivered is small, or when one studies in serial sections the very early stroma reactions.

In tumours of the skin, always, and in breast tumours whenever possible, a special mould applicator is built for each case (Fig. 1 and 2). The lesion is reproduced by means of a stent impression in a plaster cast, over which a tightly fitting stent or black-tray compound applicator is built in such a way as to be easily mounted over the part of the body, and fitting in such a way as not to allow even minimal shifts of the applicator, and so avoid the X-ray beam striking the alternate " opaque " squares of the lead chess which is recessed in the mould.

The radiation has to be delivered at right angles with the X-ray tube head applicator directed vertically. In order to avoid oblique penetration into areas beneath the opaque squares the lead chess must be in direct touch with the tumour surface. It is found in the course of treatment that owing to shrinkage of the tumour the recess sleeve in the stent mould into which the " chess " is slipped has to be filed down from time to time during the course of treatment.

The thickness of the lead, which is limited for practical considerations, permits 3 per cent of the radiation to pass through on to the protected areas. To that amount of radiation received by the not directly irradiated areas has to be added about 3–5 per cent of scatter radiation from the neighbouring exposed areas. This varies according to the size of the apertures, and decreases towards the centre of the " opaque', squares. The size of the squares has been chosen of not less than 1 cm., so as to leave an adequate amount of tissue either almost completely non-irradiated or exposed for biopsy taking.

Sections are taken before treatment is commenced and then at varying intervals. Cuts are made into the exposed and protected sectors, and also one section is made cutting across both the exposed and protected areas of tissue.

Accurate labelling of biopsy material must be insured, as no other way of recognizing the protected and exposed sectors on the histological slide is available. A working chart is found indispensable. Prior to taking the biopsy the lead mask is applied and the exposed areas are mapped out by means of gentian violet paint.

The biopsy material was fixed in sublimate-formol solution or Sousa's solution. Following paraffin embedding, sections were cut at 7$\mu$ and stained with (1) Harris' haematoxylin, eosin, (2) Van Gieson stain, (3) by Gallego's method, and (4) silver impregnation.

The irradiation was taking place every day, 5–6 days per week. The setting up was performed always by the same people, and every precaution taken in order to avoid minimal shifts.

### PRELIMINARY RESULTS.

Nine cases have been dealt with so far. Cases suitable for this investigation are not frequently encountered, especially as they have to be kept in hospital for 6–10 weeks. The critical interpretation of the slides is very difficult. Comparable areas in the protected and exposed parts have to be studied only. Two cases are described in detail:

*Case* 3.—Mrs. Emma M—, aged 67. Cancer of breast, right. Stage III. Cellular carcinoma simplex.

15.x.46 : Two years' history. General condition poor. Sprouting ulcerated growth in right breast, upper outer quadrant, fixed to pectoralis. Ulcerated area 7·5 × 6·5 × 2·5 cm., with several skin noduli scattered over breast area. Hard mass 4 × 3·5 × 3 cm. in axillary tail and hard glands in right axilla.

15.x.46 : Treatment commenced. 140 kV., 5 mA., 25 cm. F.S.D. 0·25 Cu + 1 Al. filter applicator 12 × 10 cm. Treatment through sieve, 1 cm. diam. circles. 17 treatments in 21 days. 6000r given.

16.x.46 : X-ray chest : No evidence of secondary deposits.

7.xi.46 : Treatment concluded.

Serial sections taken : (1) At commencement of treatment. (2) After 14 days ; 3800r given. (3) Three weeks after commencement of treatment; 5400 r given. (4) Section taken 7 weeks after commencement of treatment, e.g. 4 weeks after conclusion of treatment (6000r in 21 days).

No hormonal treatment was given till the middle of 1947. This was not well tolerated. Subsequently the patient had a pathological fracture in the left femur and died early in 1948 (Fig. 3, 4).

*Case* 4.—Mrs. Margaret A—, aged 64. Cancer of breast, right. Stage III. Carcinoma simplex.

12.xi.46 : Six months' history. General condition fair. Hard mass filling right breast, fixed to pectoralis and ulcerating skin in anterior axillary line over an area 8 × 5 cm. with a sloughy crater and rolled-up edges. Hard glands in right axilla. Admitted to ward. Sulphathiazole dressing ; applicator built, etc. X-ray chest : No evidence of secondary deposits.

4.xii.46 : Treatment commenced. Chess squares 2 × 2 cm., 180 kV., 10 mA. 0·5 Cu. filter, 40 cm. F.S.D. 40 r/min. Applicator 15 × 10 cm.

Sections taken : (1) Before commencement of treatment. ; (2) After 4 days ; 1500r given in 4 treatment. (3) After 14 days from commencement of treatment ; 4000r given in 14 days in 12 treatments. At this point " chess " technique abandoned and routine glancing technique reverted to. (4) Section taken when tumour received apart from the 4000r with the chess technique 2500r on each glancing field in 14 days ; 10 treatments. (5) Section taken at end of treatment (4000r to chess and 3500r on each glancing field in 5 weeks). She is remarkably well to date. When last seen, 15.xi.48, there was no sign of activity, and a small 2 sq. cm. area of granulation was healing up (Fig. 5, 6).

### GENERAL CONCLUSIONS AND INFERENCE.

From the material already available a few data can be submitted.

(1) The " sieve-chess " technique is a workable one for the study of direct and indirect effects of irradiation.

(2) The connective-tissue reaction takes place not only in the irradiated areas, but also in the neighbourhood protected from the direct irradiation.

(3) There are visible effects on cells not directly exposed to the radiation.

(4) The pattern of connective-tissue destruction and regeneration can be studied.

As a general inference it can be said that the irradiation affects the connective tissue cells and induces a kind of " over differentiation." This consists chiefly in the deposition of an excessive amount of collagenous fibrils, which are organized into solid, coarse bundles in the exposed parts where the fine connective-tissue network is grossly altered. Lymphatic channels and blood capillaries are greatly affected, compressed and blocked. This has a considerable effect on the tumour nourishment, the lack of which enhances tumour tissue degeneration.

SUMMARY.

A new method for the study of connective-tissue reaction in tumours to radiation is described, and a few preliminary data of the investigation carried out so far are given.

I am greatly indebted to Prof. R. W. Scarff, The Middlesex Hospital, for his interest in this investigation.

My thanks are due also to Dr. P. C. Koller (Chester-Beatty Institute, Royal Cancer Hospital (Free), and Dr. W. Bryan (Morbid Anatomist in this hospital), for their study of the slides and most helpful criticism. The help of Mr. E. J. Pamely (Research Technician of this Department) and Mr. C. Clarke (of the Pathological Department) is also acknowledged.

The investigation is being carried out with the aid of a grant for technical assistance from the British Empire Cancer Campaign.

REFERENCES.

ELLIS, F.—(1942) *Brit. J. Radiol.*, **15**, 348.

JOLLES, B. —(1946*a*) *Nature*, **157**, 552.—1946*b*) *Brit. J. Radiol.*, **19**, 143, 220.—(1947) *Ibid.*, **20**, 238, 405.—(1948) *Amer. J. Roentgenol.*, **60**, 745.

KOLLER, P. C., and SMITHERS, W. D.—(1946) *Brit. J. Radiol.*, **19**, 89.

MULLER, H. J.—(1940) *J. Genet.*, **11**, 1.

WINDEYER, B. W.—(1942) *Proc. Fac. Radiol.*, March.

WINDHOLZ, F.—(1947) *Radiology*, **48**, 2, 148.